# Increased sensitivity of etoposide-treated breast cancer cells with an ATM inhibitor

**DOI:** 10.1371/journal.pone.0340472

**Published:** 2026-01-20

**Authors:** Arun R. K. Kumar, Crystal Sara Shaji, Aswathi Rajagopalan, Sombodhi Bhattacharya, Wilner Martínez-López, Radha Saraswathy

**Affiliations:** 1 Biomedical Genetics Research Laboratory, School of Bio Sciences and Technology, Vellore Institute of Technology, Vellore, India; 2 Yong Loo Lin School of Medicine, National University of Singapore, Singapore; 3 Genetics Department and Biodosimetry Service, Instituto de Investigaciones Biologicas Clemente Estable, Montevideo, Uruguay; IHRC, Inc. (Human Resource Service Administration), UNITED STATES OF AMERICA

## Abstract

Breast cancer remains the leading cause of cancer-related deaths in women. Therefore, developing targeted combination therapies that improve overall survival in breast cancer patients continues to pose a major clinical challenge. Etoposide (ETO), a topoisomerase II inhibitor that induces transient double-strand breaks by blocking the cleavable complex, is currently used in high doses to treat radioresistant or metastatic breast cancers. To enhance the effectiveness of radiation and chemotherapy, targeting molecular mechanisms involved in DNA repair of induced DNA lesions could selectively increase tumor cell death. Since the effects of ETO are primarily seen during the S/G2 phases of the cell cycle, its efficacy could potentially be enhanced by using an inhibitor of a DNA repair gene involved in homologous recombination, which is mainly active during these phases. In this context, synthetic lethality refers to the concept that inhibiting or mutating two or more genes simultaneously leads to greater cell death than altering them individually. The FDA approval of Olaparib, a specific PARP inhibitor for BRCA-mutated breast cancer patients, has motivated researchers to explore other synthetic lethal interactions that could increase DNA damage accumulation, leading to cancer cell death. We demonstrate that successive treatment of breast cancer cells with specific inhibitors of ATM kinase and topoisomerase II *in vitro* can induce increased apoptosis. ATM is an apical kinase that recognizes DNA double-strand breaks and activates the homologous recombination repair pathway, either directly or through cell cycle checkpoint control. Topoisomerase II poisons generate enzyme-mediated DNA damage, leading to permanent double-strand breaks. Cytokinesis-block micronucleus assay was performed to assess the increase in DNA damage during combination treatment with inhibitors. Additionally, cell viability tests and fluorescent staining assay were conducted to evaluate the extent of cell death. We found that targeting ETO-treated breast cancer cells with an ATM kinase inhibitor, KU-55933 (KU) induced higher chromosomal damage/aberrations, as evaluated by the cytokinesis-block micronucleus assay. The ATM kinase inhibitor also significantly reduced the viability of ETO-treated breast cancer cells.

## Main

The global cancer burden has risen to 19.3 million people, according to the recent 2020 WHO report [1], making cancer one of the leading causes of death worldwide. Over the past decade, gold-standard cancer therapies, such as radiation and chemotherapy, have primarily focused on damaging tumor tissue, with little attention given to the non-targeted damage inflicted on healthy tissue by these therapies and the weakening of the patient’s immune system. The current standard treatments do not provide a long-term solution for many cancer types, often leading to cancer relapse and chemo-resistance. This highlights the growing need for more personalized, efficient treatments with fewer side effects, along with enhanced specificity in targeting and eliminating the entire cancer cell population at the tumor site, which can help improve the patient’s quality of life after diagnosis [2].

Synthetic lethality is a concept that involves identifying two or more genes which, when inhibited or knocked out together, have a significant effect on cell death, while individually targeting these genes has minimal effect on cell viability. The first FDA-approved drug for synthetic lethality, Olaparib, targets the PARP enzyme in BRCA1/2-mutant breast cancer. PARP inhibitors (PARPi) have shown promising results in clinical trials against breast cancer by directly downregulating the single-strand break repair (SSBR) pathway. BRCA1/BRCA2 proteins are crucial for the double-strand break (DSB) repair pathway. The development of PARP inhibitors has encouraged further exploration of other therapeutic targets for synthetic lethal interactions in cancers [3–7].

Topoisomerases are a family of evolutionarily conserved, essential enzymes that regulate the topological state of DNA by generating transient nicks in the DNA molecule. Their function is critical for relieving torsional stress during cellular processes such as DNA transcription, replication, repair, and chromatin remodeling [8]. Topoisomerases can relax and untangle large DNA strands, with the transient breaks they create relieving the torsional stress that develops in the DNA during replication and transcription [9]. Topoisomerase poisons are drugs that interact with the enzyme, stabilizing these transient cleavable complexes and causing single- and double-strand breaks [10,11]. Topoisomerases are classified into Topoisomerase I (Topo-I) and Topoisomerase II (Topo-II) enzymes. Topo-I enzymes create single-strand breaks, while activated Topo-II enzymes result in double-strand breaks [12,13]. Topo-II is essential for chromosome condensation and segregation during DNA replication and cell division, and complete inhibition or knockout of this enzyme can lead to cell death [9,14,15].

The reaction of Topoisomerase (Topo) with DNA produces a covalent enzyme-cleaved complex, known as the “cleavable complex,” which is a transient intermediate stabilized by chemical compounds that induce protein-associated breaks in the cell—these are known as Topoisomerase-II poisons. Etoposide, a well-known Topo-II poison, stabilizes the cleaved DNA complex, creating a fairly stable Topo-II-DNA-poison complex. This stabilization halts the re-ligation step, preventing the break from being repaired, leaving the DNA break as a recognizable double-strand break [8,16–18]. The DNA damage caused by Topo-II inhibitors is particularly pronounced during the S/G2 phase of the cell cycle.

**Fig 1:**
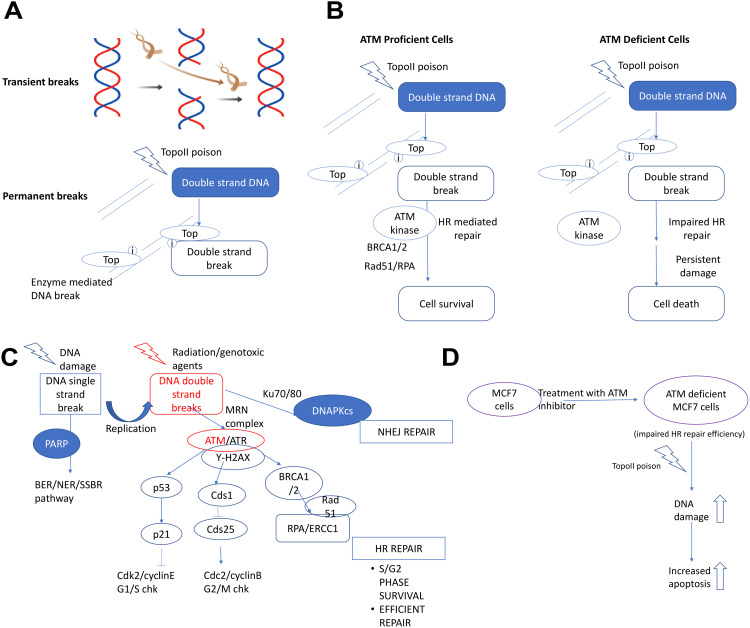
Mechanistic overview and therapeutic rationale for targeting DNA damage repair through combined Topoisomerase II poisoning and. ATM kinase inhibition in breast cancer. **(A)** Schematic representing the functions of Topoisomerase II enzyme and poison in inducing DNA damage. **(B)** Schematic of double-stranded DNA repair pathway. **(C)** Schematic of synthetic lethality in breast cancer between Topoisomerase-II and ATM kinase (with Topoisomerase-II poison and ATM inhibition). **(D)** Schematic of the hypothesis involving the combinational treatment against breast cancer.

Topo-II poisons can cause direct double-strand DNA breaks or indirectly induce them when single-strand breaks encounter a replication fork (**Fig 1A**). Topo-II poisons bind to the enzyme, forming a Topo-II-DNA-poison complex that creates a double-strand nick but prevents the enzyme from re-ligating the break. Two major pathways are responsible for repairing these double-stranded breaks: Non-homologous end joining (NHEJ) and Homologous recombination repair (HRR). HRR is a high-fidelity repair mechanism that uses a sister chromatid as a template and is predominantly active during the S/G2 phases of the cell cycle. In contrast, the NHEJ repair pathway is more error-prone, potentially losing up to 20 nucleotides during the repair process [19]. The HRR pathway promotes lesion substitution primarily during the S and G2 phases of the cell cycle, and it is closely associated with the S and G2 checkpoints. An overview of the proteins involved in these checkpoints is depicted in **Fig 1B**. The HRR pathway is responsible for repairing collapsed replication forks, which promote double-strand damage from single-stranded lesions. Due to its high fidelity, which uses a complementary DNA strand as a template, HRR plays a crucial role in maintaining genomic stability.

Several synthetic lethal targets that involve interactions between ATM kinase, ATR, and other DNA damage response (DDR) components are being actively investigated. PARP inhibition has shown lethal activity in ATM-deficient cells [20–22]. Research on non-small cell lung cancer (NSCLC) cells with non-functional p53 and ATM kinase has also demonstrated high sensitivity to ATR inhibition [23,24]. Additionally, inhibition or knockdown of ATM has been found to be synthetically lethal in cells with FA pathway deficiencies [25,26]. The efficiency of a cell’s DNA repair system plays a critical role in cancer cells’ ability to evade apoptosis, a key survival mechanism. A defective DNA repair system increases the likelihood of mutations and genetic instability, both of which contribute to cancer progression. Veuger and Curtin suggest that redundancy exists within DNA repair systems, such that the loss of one repair pathway may impair the cell’s response to DNA damage, either partially or entirely [27]. This redundancy allows cancer cells to rely on backup repair mechanisms to maintain cellular integrity. Inherited deficiencies in the homologous recombination repair (HRR) pathway are associated with a higher risk of developing cancers [28]. Carriers of these defective genes have one functional allele, and cancer development depends on the somatic inactivation of the second allele, which impairs the cell’s ability to repair DNA. Thus, the efficacy of cancer treatment is closely tied to the functionality of DNA repair mechanisms. Increased DNA repair activity can lead to the removal of DNA damage and increased resistance to therapy, while defects in mismatch repair (MMR) can also lead to therapy resistance [29]. Conversely, non-functional HRR in tumors makes them more susceptible to DNA cross-linking agents [30]. Epigenetic silencing of ATM has been associated with cancers such as lung, breast, colorectal, and head and neck squamous cell carcinomas [27,31,32].

A promising approach in cancer therapy involves inhibiting DNA repair pathways that respond to double-strand breaks (DSBs), as these pathways play a key role in cancer cell survival. DSB repair inhibitors can reduce the tumor’s ability to respond to DNA damage, leading to cell cycle arrest and apoptosis (**Fig 1C**). These inhibitors can also enhance the effectiveness of radiotherapy and chemotherapy by overcoming resistance. Removing therapy resistance creates an imbalance in the DDR, which can be exploited to target cancer cells. Supplementing treatment with a DSB-inducing agent, such as Topo-II poisons, increases the production of DSBs. Therefore, we propose a mechanism in which a combination therapy using the ATM kinase inhibitor (KU-55933 or KU) to inhibit DSB repair, along with a Topo-II poison to induce DSBs, could target breast cancer cells based on its theoretical potential (**Fig 1D**). We hypothesize that dysregulation of the DSB repair pathway will increase cell cycle arrest and, due to repair inefficiency, lead to apoptosis.

## Methods

### Reagents and drug formulations

KU-55933 (KU), Sigma was used as ATM inhibitor and ETO (Sigma) was used as Topoisomerase II poison in the experiments. To induce DNA double-strand damage in MCF cells, ETO and KU were dissolved in DMSO stock and diluted to appropriate concentrations in media for the experimental design with final DMSO concentrations less than 0.1%. Dimethyl sulfoxide (DMSO) and Chloroform were purchased from Fisher Scientific, India. DAPI (Sigma Ltd, India), MTT (Sigma), Methanol (HPLC grade), glacial acetic acid were also purchased for the experiments. The following antibodies mouse anti-phospho-ATM (Ser-1981), mouse anti-GAPDH and Goat anti-mouse IgG (H + L) secondary antibody were purchased from Proteintech.

### Cell culture

Breast adenocarcinoma cell line of Caucasian origin, MCF-7 were used for the experiments. The cells were cultured in Dulbecco’s Modified Eagle’s Medium (DMEM) containing L-glutamine (Sigma Ltd, India) supplemented with 1% Penicillin-Streptomycin (PenStrep) antimycotic solution (HiMedia Ltd, India) and 10% foetal bovine serum (HiMedia Ltd, India). Cells were maintained at 37^0^C in a water-jacketed humidified 5% CO_2_ incubator. Cells were subcultured every three days into new cell culture flasks. MCF-7 were seeded 24 hours before the treatment at a concentration of 4–5 X 10^4^ cells/ml. In individual treatments, the cells were exposed or unexposed to KU or ETO for a designated amount of time. For combinational treatments, cells were either, 1. pre-treated with KU for 2 hours followed by 2 hours of ETO treatment, or 2. pre-treated with ETO for 2 hours followed by 2 hours of KU treatment, or 3. treated together (ETO and KU) for 2 hours. After appropriate treatments, cells were washed to remove excess drug and analysed for downstream experiments.

### Morphological change to ETO treatment

MCF-7 cells were seeded in two 60 mm cell culture plates at 10^6^ cells in each plate 24 hours before treatment. The plates were assigned either as an untreated control or treated with ETO at a concentration of 20 µM for 6 hours in 37°C; 5% CO2 conditions. Later, the plates were observed under an inverted microscope and images captured.

### Cytokinesis block micronucleus test

To quantify the DNA damage or genetic instability in the cells a cytogenetic technique, Cytokinesis block micronucleus assay was performed. ETO was used as Topoisomerase II poison and to create double-strand DNA damage and KU inhibits the activation of ATM kinase in the cells. Cytochalasin B (Sigma) was used as cytokinesis block agent. Cells were seeded 20 hours before treatment at a concentration of 2.5 x 10^5^ cells per flask. They were treated with the chemotherapy drugs individually or in combination in for 2 hours each. After treatments, the media was removed, and the cells were washed twice with 1X PBS. Then, the cells were replenished with new media containing Cytochalasin-B (6ug/ml) and incubated for another 48 hours to block cytokinesis in the next cell division. At 72 hours since seeding, the cells were recovered and fixed with freshly prepared fixative containing methanol: acetic acid (3:1) with 1% formaldehyde. A wash step was followed after 1 hour with methanol: acetic acid (3:1) without formaldehyde. Slides were prepared by gently dropping the sample and air drying. Slides were then stained with Giemsa (10%) in PBS (HiMedia Ltd, India) followed by 3x washes in PBS to remove excess stain and the slides were air dried.

The counting was performed in a blinded manner. The slides were observed under a light microscope (Labomed, Olympus) and images were captured using Olympus fluorescence microscope software. At least thousand binucleates were counted per sample with technical triplicates and biological replicates. The data obtained contained the count of binucleates, multi-nucleates, micronuclei in binucleated cells, apoptotic, necrotic cells, nuclear bud and nucleoplasmic bridges.

### Western blotting

MCF-7 cells were seeded into different plates at the concentration of 2 x 10^6^ cells and were treated for 6 hours with ETO. After the treatments, the cells were washed with 1X PBS and trypsinised to obtain the cell pellet. The cell extracts were obtained using RIPA lysis buffer containing protease and phosphatase inhibitor mix (ThermoFisher Scientific). The cell extracts were incubated on ice for at least 20 minutes and then centrifuged at 14000g for 10 minutes – 4 ^0^C. The protein content of the supernatants was measured using a Nanodrop spectrophotometer. Protein samples were mixed with 4X Laemmli loading buﬀer, DTT and heated at 100°C for 10 minutes. Then, equal proteins were loaded onto a precast 10% Tris Glycine gel (Biorad). The proteins were separated in 1X SDS Tris Glycine running buﬀer. The proteins were then transferred onto a PVDF membrane (Merck, India) using a wet transfer system. The membrane was washed with Milli-Q and blocked with 5% non-fat dry milk/BSA for 1 hour. Immunoblotting was performed using mouse anti-human phospho-ATM (Ser-1981), mouse anti-human GAPDH followed with incubation of Goat anti-mouse IgG (H + L) secondary antibody (Proteintech) at recommended product instructions. The excess antibody was removed by intermittent washing with 1X Tris Buffered Saline with 0.2% Tween-20 (TBST – with or without sodium fluoride) thrice (10 minutes each). The membrane was exposed with ECL chemiluminescent reagents (ThermoFisher Scientific) as per the instruction manual and images were captured under a Cytiva CCD imager unit. Quantifications of protein expression were performed using Image J software (National Institute of Health USA, Version 1.52a). Data are expressed as mean ± S.D. relative to untreated samples. Equal loading was verified using a loading control GAPDH expression in corresponding gels.

### MTT assay

Cell viability was evaluated using MTT reagent[3-(4,5-dimethylthiazol-2-yl)-2,5-diphenyltetrazolium bromide]. The cells were seeded equally into flat bottom, transparent, 96 well tissue culture plates 24 hours before the treatments at a concentration of 10^4^ cells/well and maintained in 37°C/5% CO2 incubator. The cells were then treated as per the experimental design for increasing concentrations of the chemotherapy drugs. After the treatments, the media mixed with drugs were removed and renewed with fresh media mixed with MTT (0.5 mg/ml) and maintained at 37°C;5% CO2 conditions for 4 hours. The purple precipitated formazan crystals were solubilised with 100µl DMSO. The luminescent optical densitometric reading at 570nm was measured using a UV-vis spectrophotometer microplate reader with installed software (Bio-Rad Ltd, India). The data represented are a mean of at least three wells and the experiment was analysed three times to gain higher confidence. Cell viability was evaluated as (treatment OD/control OD) × 100, where treatment OD represents the optical density values of treated cells and control OD represents the optical density values of control untreated cells.

### Apoptotic triple fluorescent staining

Apoptotic cell population was counted using the differential fluorescent stains such as Propidium iodide (PI) (Invitrogen), Fluorescein diacetate (FDA) (Invitrogen) and Hoechst 33342 (HO) (Invitrogen). PI and HO are nuclear stains that function by attaching to the DNA provided the nuclear membrane is intact in the cell, denoting cellular viability. FDA stains the cytoplasm green in viable cells having an intact membrane.

The stains are solubilised with diluents into the stock concentrations as mentioned in the manufacturers sheet. Prepare a 100 μl of working fluorochrome solution by mixing 20 μl of FDA, 20 μl of HO and 10 μl of PI in 50 μl PBS. MCF-7 cells were seeded at concentration of 10000 cells per flask for the different experiments 24 hours before the treatments. The cells are treated with the chemotherapy drugs for 6h, 12h or 48h. After the treatments, the cells were washed with 1X PBS. They are trypsinised and centrifuged at 1500 rpm for 5 minutes. The supernatant is discarded and the cell pellet is resuspended in 400 μl of PBS. 7 µl of the fluorochrome to the cells and incubated in the dark for a minute. Clean glass slides are taken and mounted with required sample and are covered with coverslips without air bubbles. They are observed under a fluorescent microscope fitted with long-pass filters to differentiate the different excitations of the triple dyes (Olympus). The data represents the cells based on the proportion of live, apoptotic and necrotic cell population in different treatments.

### Statistical analysis

Student’s t tests and one way ANOVA with post-test using Tukey’s multiple comparisons tests were performed to compare means of each column with every other column. Analysed data are represented in terms of mean value ± S.D. unless specified otherwise.

## Results

### ETO exposure changes the morphological structure of MCF-7 breast cancer cells

**Fig 2:**
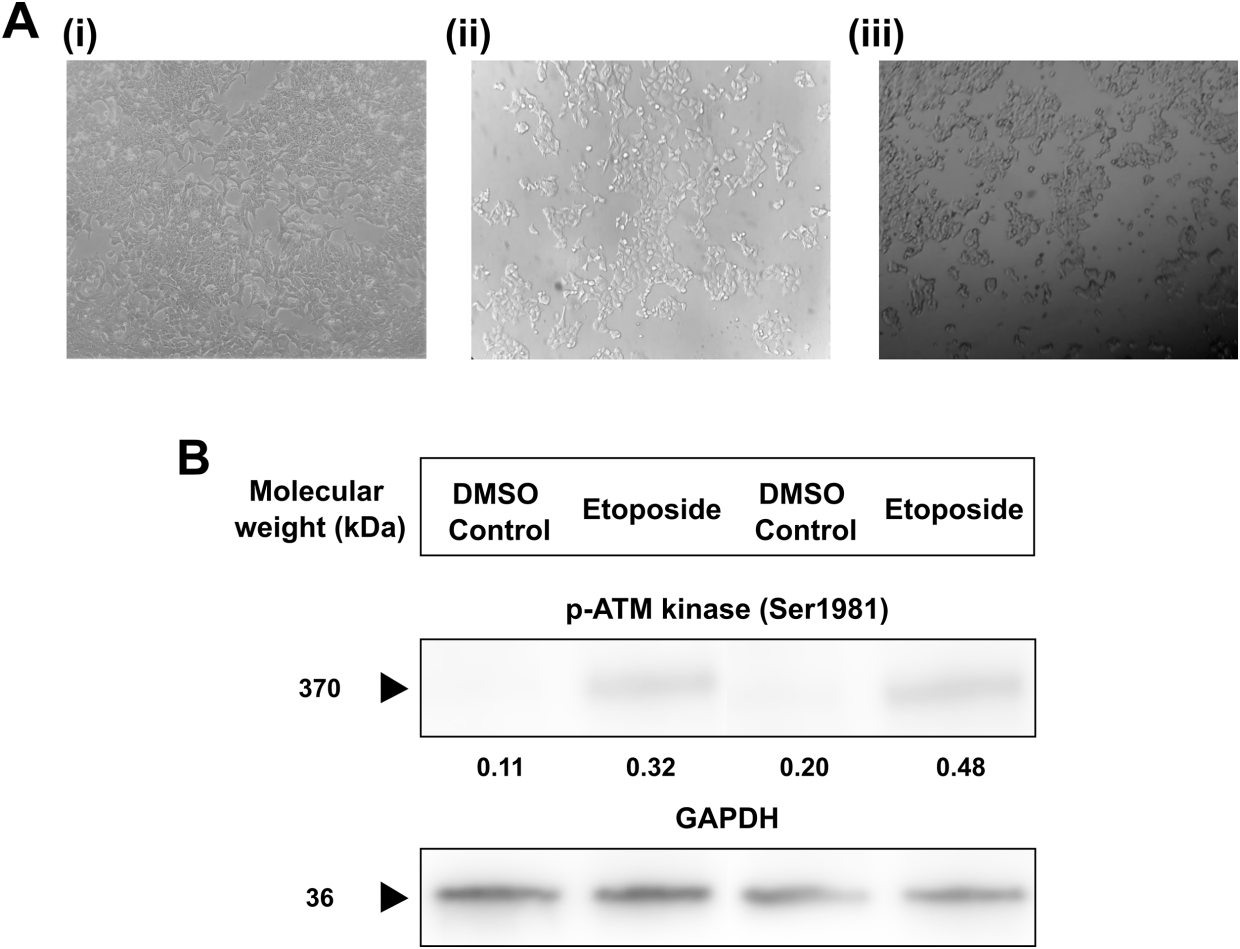
ETO exposure induces morphological changes and expression of p-ATM kinase in breast cancer cells. **(A)** Morphological changes in MCF-7 cells on **(i)** Untreated cells, **(ii)** ETO (20 µM) exposure for 6h, **(iii)** ETO (20 µM) exposure for 18 hours observed under inverted microscope. During exposure to ETO breast cancer cells were qualitative analysed and morphological representations are presented. **(B)** Western blotting for phosphorylated-ATM kinase (p-ATM) after addition of ETO (20 µM) for 6 hours. The representative western blot densitometry plots were quantified using Image J across four biological replicates and presented as ratio of intensities of p-ATM and GAPDH (mean intensity).

Breast adenocarcinoma MCF-7 are of epithelial cell origin which has a unique fibroblast nature in culture. It can be visualised when observed under an inverted microscope. Etoposide exposure induced notable morphological alterations in MCF-7 cells compared to the untreated control: treated cells appeared rounded, shrunken, and partially detached, and a population of small, opaque, floating cells was observed (representative images shown in **Fig 2A**). These observations are qualitative and were not accompanied by direct morphological counts in the current experiments.

### ETO exposure to breast cancer cells increases the expression status of p-ATM kinase

The expression of p-ATM kinase (Ser 1981) was observed when ETO treatment was given to breast cancer cells. ETO (20 µM) concentration for 2 hour was able to induce the phosphorylation and activation of p-ATM kinase in breast cancer cells suggesting that the DNA repair is initiated in the cells after ETO treatment and is still functional in the breast cancer cells. (**Fig 2B**) The quantified mean intensities of DMSO Control and ETO treatment between four different biological repetitions were **0.18 ± 0.14** and **0.37 ± 0.07** respectively.

### ETO induces genomic instability and DNA damage in the breast cancer cells

**Fig 3:**
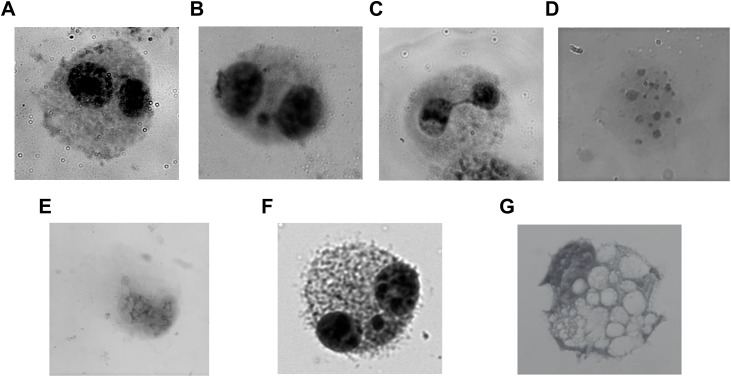
Representative images obtained from CBMN assay: (A) Binucleate cell, (B) Binucleate with a micronucleus (MNi), (C) Binucleate with a nucleoplasmic bridge (NPB), (D) Apoptotic cell, (E) Binucleate with a nuclear bud (NBud), (F) Multinucleate cell, (G) Necrotic cell.

Genomic instability is a clear indication of DNA damage that is induced in the cells due to DNA damage agents. ETO acts as a Topo-II poison and creates instant DNA double-strand DNA damage that is expressed as chromosomal damage during CBMN assay. The chromosomal damage can be visualised in the form of micronuclei (MNi), nucleoplasmic bridges (NPBs) or nuclear buds (NBuds) in the cells. Micronuclei are a result of DNA/chromosomal damage resulting in chromosomal breaks, while NPBs represent dicentric chromosomes and buds a resultant of altered abnormal gene amplification due to the damage inducing agent (**Fig 3**). Cytokinesis was blocked using Cytochalasin-B (6 µg/ml) which ends in the nuclear division possible but the cell remains undivided. So, when the cells containing damaged DNA divides the damage is expressed as the chromosomal damage.

**Fig 4:**
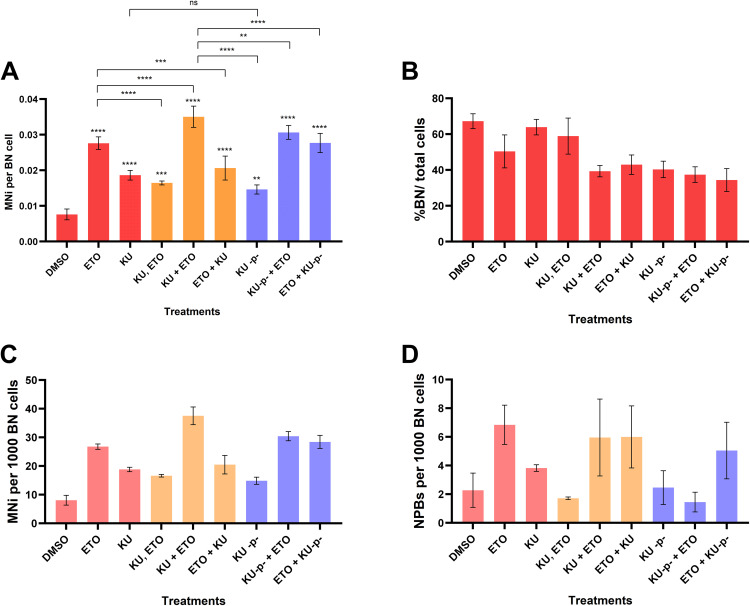
Cytokinesis Block Micronuclei Assay shows DNA damage after combinational treatment. **(A)** DNA damage inducing agents like ETO induces a significant damage increase in resulting in micronuclei and a higher MNi/BN cell ratio. Combination treatment (KU_ETO) induced higher DNA damage compared with other combinational treatments, including prolonged ATM inhibition (KU^-p-^ + ETO and ETO + KU^-p-^) suggesting short durations of ATM block can induce higher cancer cell death. **(B)** % binucleate (BN) cells/total cells counted or CBPI index. The binucleate cell population remained similar during individual treatments (50-65% of the total cells) and decreased to a 35-45% binucleate percentage in the combinational treatments. The data used to create this CBMN graph is attached in S1 Table. **(C-D)** Frequency of micronuclei (MNi) per 1000 BN cells and frequency of nuclear buds (NPBs) per 1000 BN cells counted. MNi and NPBs are significantly higher in ETO and combinational treatments compared with controls in MCF7 cells. Data represented as Mean ±SD. Statistical analyses performed with one way ANOVA, Student’s T-test (unpaired) with post-tests using Tukey’s multiple comparisons tests. *p < 0.05, **p < 0.01, ***p < 0.001, ****p < 0.0001. The data used to create this CBMN plots is attached in S2 Table.

The data collected represented the frequency of binucleates (BN), multinucleates, micronuclei observed in binucleates (MNi), nucleoplasmic bridges in BN cells (NPBs), nuclear buds in BN cells, apoptotic and necrotic cells. The data set for the micronucleus test counting is included in the table (**S1 Table**) and represented in **S1 Fig**. The graph % BN/total cells counted represents that the binucleate count was fairly the same during the course of different combinational treatments. Exposure to ETO induced significant MNi and NPBs formation in the breast cancer cells. Furthermore, The combination treatment involving KU + ETO was clearly inducing more DNA damage than other treatment points indicating that DNA repair deficiency causes more chromosomal damage that converts more cells to drive into apoptosis. Prolonging the ATM deficiency (represented as KU^-p-^) also seemed to cause a higher genomic instability in breast cancer observed as higher count of micronuclei in the treatments, KU^-p-^ + ETO and ETO + KU^-p-^. Notably, Combinational treatment with pre-treatment with KU before ETO (KU + ETO) induced significantly more damage than other treatments such as pre-treatment with ETO before KU (ETO + KU). While prolonged exposure to ATM inhibitor did not significantly increase the MNi (KU^-p-^ + ETO, ETO + KU^-p-^). This suggests that the suppresion of ATM kinase might be responsible for the blocked DNA repair of ETO induced DNA damage. (**Fig 4**)

### ATM inhibition decreases the viability of breast cancer cells exposed to ETO treatment

The cell viability of breast cancer cells was analysed using MTT assay. It was used to determine the dosage needed for ETO and KU treatments. Thus, a dose dependent exposure of the drugs to the cells were conducted for a designated 24 hours. From prior investigations conducted by our group, the concentrations chosen were 0–40 µM.

**Fig 5:**
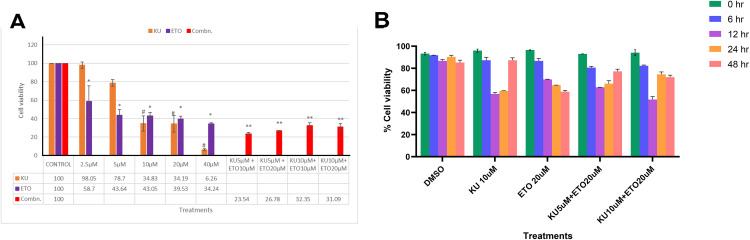
Cell viability is affected by exposure to ETO in ATM inhibited breast cancer cells. (A) Dose dependent study of cell viability for the combinational treatments We observe that individual treatments of KU with breast cancer cells can reduce the viability up to 6% while ETO on the other hand reduced the viability to 34%. As the KU can induce toxicity, we decided to use 5 µM concentration of KU for later assays. The viability of combination can be observed to be around 20-40% in the breast cancer cells. (B) Time dependent cell viability for the combinational treatments between 6–48 hours. 2 hours treatment of KU or ETO does not affect the viability more than 50%. But the exposure of 2h is enough to induce immediate DNA double strand damage (observed via CBMN). Beyond 12h, the cells recuperate and increase in viability which could be due to the temporary inhibition of ATM by KU addition. This could explain the insignificant viability for the combinational treatments. Legend: – KU treatment (5 µM or 10 µM), ETO treatment (10 µM or 20 µM), Combinational treatment KU (5-10 µM) +ETO (10-20 µM). Data represented as Mean ±SD. Statistical analyses performed with one way ANOVA, Student’s t-test (unpaired) with post-tests using Tukey’s multiple comparisons tests. *p < 0.05, **p < 0.01, ***p < 0.001, ****p < 0.0001.

Individual treatment of KU and ETO for 24 hours in MCF7 cells resulted in significant reduction in cell viability. Significant reduction in the viability of breast cancer cells was seen at concentrations higher than 10 µM KU for 24 hours, while effect on diminishing the cell viability with ETO was observed at concentrations as small as 2.5–5 µM. Viability was reduced to 6.26% with 40 µM KU exposure and 34.24% with treatment with 40 µM ETO. Combinational targeting was also performed, and the data showed tremendous increase in the dead cell population as high as 75% in treatments KU 5 µM + ETO 10 µM condition (**Fig 5A**). A time dependent assay revealed that the viability of cells during combinational treatment beyond 24 hours was slightly higher which could be due to the temporary inhibition of ATM by KU addition (**Fig 5B**).

### Combinatorial therapy increases the apoptotic cell population in breast cancer

Triple staining of the cells was used to quantify the increase in apoptotic cells during the combination treatment of ETO and KU. The concentrations of ETO and KU drugs were determined as 20 µM and 5–10 µM respectively from a previous pilot study conducted using cell viability assays. The triple fluorescent stains used were FDA, HO and PI. FDA absorbs light at blue wavelength and emits a green colour. It has a permeability in living as well as dead cells. It stains the cytoplasm of live round cells green and dead cells have an irregular shaped light green colour. PI nuclear stain absorbs green light and emits red colour. PI has a permeability only in dead cells. HO stains nucleolus and nucleus in blue by absorbing UV light. HO stains the nucleus of both live and fixed/dead cells. So, adding triple dyes to the cells, the data can be gathered with three steps. Cells with cytoplasm green and nucleus blue are living cells. Cells with pale green cytoplasm and strong blue nuclear signal with fragments are apoptotic cells. Cells with PI are necrotic cells. Late apoptotic cells have an irregular fragmented nucleus stained with red PI. The data collected arranged the cells into four categories, ‘Live Cells’, ‘Apoptotic Cells’ or ‘Necrotic Cells’.

**Fig 6:**
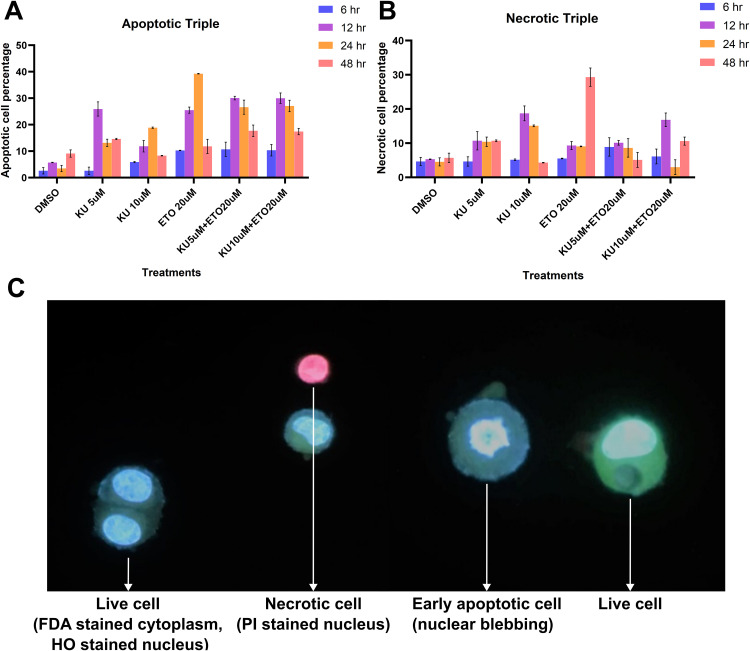
Increase in apoptotic and necrotic cells monitored through Triple fluorescent staining assay. (A) Percentile of the Apoptotic and (B) Necrotic cell population during KU, ETO and combinational treatments in MCF-7 cells for the designated periods. There is an increasing trend observed in the apoptotic population during ETO treatments and the combinational treatments. Notable significant increase in observed in the apoptotic cells in ETO and combination treatments in 12h and 24h time points. Observation of 48h shows lower apoptotic cell death compared to previous time points. For necrotic cell count, there is no significant difference in the treatment and time points. The high necrosis in KU-10 µM and ETO-20 µM could be due to the delayed involvement of PARP mediated necrosis or other p53 independent pathways. (C) representative images of Live, early and late apoptotic, and necrotic cells observed during Triple staining. Legends: FDA – Fluorescein Diacetate, HO – Hoechst 33342, PI – Propidium Iodide. Quantified data can be found in S3 Table and S4 Table.[Subxref3].

The increase of the apoptotic and necrotic cell population was observed in MCF-7 cells. A significant increase in apoptosis was marked in the combinational treatment involving KU + ETO at 12 hours and 24 hours. represented in (**Fig 6A**). This must be resulting due to the effect of the drugs in escalating the DNA damage. ETO and KU rend to increase the cell death at a higher time of exposure such as 12 hours and 24 hours owing to the downstream effectors associated with ETO and the promotion of DNA repair deficiency that leads to apoptosis in the cells. Necrotic cells were also observed in the treatments expressing the stress caused by the drugs in the cells shown in **Fig 6B**. Representative images captured during the staining experiment are shown in **Fig 6C**.

## Discussion

The breast cancer MCF-7 cells that are deficient in ATM kinase with KU addition are sensitised by the DNA damage induced by Topoisomerase-II poison ETO. Exceptional biomedical approaches are remodelling cancer research involving double-strand break repair pathway exploiting synthetic lethality. Loss of ATM and p53 sensitises mouse embryonic fibroblasts to Topo-II poisons and other metabolites. There was an observed 2–4 fold increase in loss of cell viability in the combinational inhibition therapy [33]. Further articles have delineated the use of KU inhibiting ATM kinase and sensitising cells to radiation and chemotherapy [34–37]. Similarly, we observed a combinational cell death to 80% from 22% in individual treatments. Following the induction of DNA damage, the initiation of apoptosis was observed through apoptotic cells by fluorescent staining, and loss of viability by MTT. Using genomic wide associations that could decipher the links between genetic and epigenetic patterns and clonal design that promote the key characteristics of tumour tissue [38].

ETO exposure induces double-strand breaks (DSBs), leading to genomic instability in breast cells. When ATM is inhibited prior to ETO exposure, the resulting ATM deficiency increases genetic damage, indicating that the loss of ATM hinders homologous recombination repair (HRR) and allows ETO-induced DSBs to accumulate. Unrepaired damage eventually progresses to apoptosis, as evidenced by p53 activation. This suggests potential synthetic lethal interactions between Topoisomerase-II and ATM kinase, offering a therapeutic strategy to target ATM-deficient cancers. Targeting DNA repair-deficient cancers, such as those found in leukemia, mantle cell lymphoma, breast, and lung cancers, by exposing them to Topo-II poisons could be a promising approach. However, further studies are needed to better understand these complex pathways and identify suitable treatments [39,40].

A limitation of the present study is the lack of quantitative morphometric analysis for the microscopy observations. While reduced viability was observed (**Fig 6A**), we did not perform systematic counts of floating vs. adherent cells during these experiments. Future work will include hemocytometer-based counts and automated image analysis to precisely quantify detachment and rounding.

Another limitation of this study is that all experiments were performed *in vitro* using a single breast cancer cell line (MCF-7). While these data provide important mechanistic insights into the interaction between ATM inhibition and Topo-II induced DNA damage, they may not fully reflect the complexity of tumor microenvironments or pharmacokinetics *in vivo*. The selectivity and potential cytotoxicity of KU-55933 toward non-tumorigenic cells were not evaluated in this study; testing in non-tumorigenic breast epithelial cells, such as MCF-10A, would be valuable to determine whether the observed effects are specific to malignant cells. Future work will also include *in vivo* validation using xenograft or orthotopic breast tumor models to assess whether combinational ATM and Topo-II inhibition can achieve selective tumor killing without affecting normal tissue. Such studies will help to establish the translational relevance and safety of this therapeutic strategy.

The findings of this study provide a proof of concept that pharmacological inhibition of ATM can enhance the cytotoxicity of Topo-II poisons such as Etoposide in breast cancer cells, supporting the rationale for targeting the DNA damage response as a therapeutic strategy. Translationally, combining ATM inhibitors with DNA-damaging chemotherapies or radiotherapy could potentiate tumor-specific apoptosis by exploiting defective repair pathways often found in cancer cells. Although KU-55933 is a well-established and selective ATP-competitive inhibitor of ATM kinase, its use in cell-based studies is associated with several experimental constraints.

First, KU-55933 exhibits poor solubility and limited stability in aqueous media, often requiring high concentrations of DMSO for dissolution, which may introduce solvent-related cytotoxicity or off-target effects. Second, while it displays strong in vitro selectivity for ATM, off-target inhibition of related kinases such as ATR or DNA-PKcs has been reported at higher doses, potentially confounding interpretations of DNA damage response outcomes.

Subsequent analogs such as KU-60019 and AZD0156 have shown improved potency and pharmacological profiles, suggesting more feasible options for preclinical and clinical development. Future work should therefore focus on evaluating complete ATM knockout models or newer-generation inhibitors, in combination with Topo-II poisons, to assess both therapeutic efficacy and systemic toxicity.

Additionally, the slight increase in cell viability observed during combinational treatment beyond 12 hours may be attributed to the transient nature of ATM inhibition by KU-55933 (**Fig 5B**). KU-55933 is a reversible ATP-competitive inhibitor whose effect diminishes over time due to metabolic degradation or reduced intracellular retention. As ATM kinase activity partially recovers, the DNA damage response (DDR) may be reactivated, allowing limited repair of double-strand breaks and transient restoration of cell viability. This temporal recovery highlights the importance of sustained DDR suppression to maximize cytotoxicity during combination therapy.

To further validate this mechanism, future studies will employ ATM-deficient or ATM-knockout breast cancer cell lines to confirm that the observed sensitization to Etoposide arises specifically from ATM loss-of-function. Comparing KU-55933–treated wild-type cells with ATM-null cells will help distinguish pharmacologic off-target effects from direct consequences of ATM inhibition and provide stronger mechanistic evidence supporting the proposed model.

Given the limitations of transient ATM kinase inhibition, alternative approaches such as genetic silencing or CRISPR-based knockout of ATM could offer more precise mechanistic validation. Recently, non-viral nanostraw-mediated transfection has emerged as a promising platform for efficient and minimally invasive gene delivery [41].

In summary, KU is a potent inhibitor of ATM kinase. Combining KU with ETO significantly increased cancer cell sensitivity to treatment, with even more pronounced effects when both inhibitors were used together. This suggests potential for improving treatment outcomes in solid breast cancer, especially in patients with defects in DNA repair pathways like BRCA or ATM mutations. Targeting one repair pathway when the other is defective could maximize treatment effectiveness with minimal toxicity.

Extensive research is being conducted on the DNA damage response (DDR) and the role of ATM kinase to better understand its downstream components in maintaining a cell’s normal microenvironment and homeostasis. Gaining deeper insights into ATM’s relationship with mTOR and PI3K/Akt signaling pathways could support the use of pathway inhibitors in cancer therapy. It may also shed light on cellular communication in cancer and diseases like ataxia-telangiectasia. Our data shows the recruitment of DDR components to damage sites and the repair of double-strand breaks (DSBs). Further investigation is needed to explore the pharmacological properties of these inhibitors in preclinical and clinical trials, with the potential for market availability in the near future. Significant progress has been made in targeting proteins such as PARP, BRCA1/2, CHK1/2, DNA-PKcs, and ATR. We propose that the ATM inhibitor KU, in combination with the Topoisomerase-II poison ETO, increases DNA damage and promotes apoptotic cell death. Additional preclinical models and clinical trials are necessary to validate and support the synthetic lethal interactions between Topoisomerase-II and ATM kinase.

### Highlights

- Combinational treatment with inhibitors of ATM kinase and Topoisomerase II cause apoptosis.
- CBMN assay on MCF-7 cells shows increase in DNA damage in combinational treatment.
- Cell viability assessed using Triple staining and MTT assay
- Western blotting for assessing the expression of p-ATM kinase

## Supporting information

S1 Table Summary table showing the % BN cells out of total cells for each treatment point during cytokinesis-block micronucleus assay. Data from this table was used to plot Fig 4.
(PDF)

S2 Table Summary of all the treatment points during cytokinesis-block micronucleus assay. Data from this table was used to plot Fig 4 and S1 Fig.
(PDF)

S3 Table Summary of apoptotic cells observed after different treatment points during Triple staining of cells with Hoechst 33342 (HO), Propidium Iodide (PI) and Fluorescein diacetate (FDA). Data from this table was used to plot Fig 6A. (PDF)

S4 Table Summary of necrotic cells observed after different treatment points during Triple staining of cells with Hoechst 33342 (HO), Propidium Iodide (PI) and Fluorescein diacetate (FDA). Data from this table was used to plot Fig 6B. (PDF)

S1 Fig Summary pie charts showing the frequency of all CBMN endpoints across treatment groups. (PDF)
