# Supplementary material for: Increased sensitivity of etoposide-treated breast cancer cells with an ATM inhibitor
Source: PLoS One. 2026 Jan 20;21(1):e0340472. doi: 10.1371/journal.pone.0340472 (PMC12818603; doi:10.1371/journal.pone.0340472)
Supplement: S1 Table — Data from this table was used to plot Fig 4. (PDF) [file pone.0340472.s001.pdf]

Supplementary Material for

Increased sensitivity of etoposide-treated breast cancer cells with an ATM inhibitor

Arun R. K. Kumar [1,2] , Crystal Sara Shaji [1] , Aswathi R [1], Sombodhi Bhattacharya [1], Wilner Martínez-López [1,3] , Radha Saraswathy* [1]

[1] Biomedical Genetics Research Laboratory, School of Bio Sciences and Technology, Vellore Institute of Technology, Vellore, India

[2] Yong Loo Lin School of Medicine, National University of Singapore, Singapore 117597, Singapore

[3] Genetics Department and Biodosimetry Service, Instituto de Investigaciones Biologicas Clemente Estable, Montevideo, Uruguay

Supplementary Tables

S1 Table: Summary table showing the % BN cells out of total cells for each treatment point during cytokinesis-block micronucleus assay. Data from this table was used to plot Fig 4.

Treatment	%BN/ total cells	std. dev
DMSO	67.21	4.13
ETO	50.37	9.22
KU	63.91	4.34
KU^{-p-}	58.89	10.07
KU/ETO	39.31	3.16
KU+ETO	42.95	5.43
KU^{-p-} + ETO	40.28	4.58
ETO+KU	37.39	4.34
ETO + KU^{-p-}	34.38	6.38

ETO – Etoposide, KU – KU-55933, -p- - prolonged addition of the drug in the culture (representing complete ATM kinase inhibition)
