# Supplementary material for: Increased sensitivity of etoposide-treated breast cancer cells with an ATM inhibitor
Source: PLoS One. 2026 Jan 20;21(1):e0340472. doi: 10.1371/journal.pone.0340472 (PMC12818603; doi:10.1371/journal.pone.0340472)
Supplement: S2 Table — Data from this table was used to plot Fig 4 and S1 Fig. (PDF) [file pone.0340472.s002.pdf]

S2 Table: Summary of all the treatment points during cytokinesis-block micronucleus assay. Data from this table was used to plot Fig 4 and S1 Figure.

Treatment	Total MNi counted	total BN cells counted	total NPBs in BN cells	total Nbuds in BN cells	total cells counted
DMSO	86	11320	22	9	16842
ETOPOSIDE	167	6054	43	5	12019
KU	98	5266	17	5	8239
KU/ETO	99	6022	14	12	15318
KU+ETO	335	9564	13	5	22264
ETO+KU	117	5677	66	18	15180
KU^{-p-}	74	5067	5	6	8604
KU^{-p-} + ETO	104	3395	35	5	8427
ETO + KU^{-p-}	218	7879	34	13	22914

ETO – Etoposide, KU – KU-55933, -p- - prolonged addition of the drug in the culture (representing complete ATM kinase inhibition)
