# Supplementary material for: Increased sensitivity of etoposide-treated breast cancer cells with an ATM inhibitor
Source: PLoS One. 2026 Jan 20;21(1):e0340472. doi: 10.1371/journal.pone.0340472 (PMC12818603; doi:10.1371/journal.pone.0340472)
Supplement: S3 Table — (PDF) [file pone.0340472.s003.pdf]

S3 Table: Summary of apoptotic cells observed after different treatment points during Triple staining of cells with Hoechst 33342 (HO), Propidium Iodide (PI) and Fluorescein diacetate (FDA). Data from this table was used to plot Fig 6A.

**Treatment**	**% Apoptotic cells at 6 hours**	**% Apoptotic cells at 12 hours**	**% Apoptotic cells at 24 hours**	**% Apoptotic cells at 48 hours**
**DMSO**	2.609701	5.7	3.459701	9.1
**KU 5uM**	2.609701	25.9	13.16	14.6
**KU 10uM**	5.879268	11.85	18.85927	8.3
**ETO 20uM**	10.27679	25.47	39.24791	11.8
**KU5uM+ETO20uM**	10.69332	30.05	26.60299	17.7
