# Supplementary material for: Increased sensitivity of etoposide-treated breast cancer cells with an ATM inhibitor
Source: PLoS One. 2026 Jan 20;21(1):e0340472. doi: 10.1371/journal.pone.0340472 (PMC12818603; doi:10.1371/journal.pone.0340472)
Supplement: S4 Table — (PDF) [file pone.0340472.s004.pdf]

S4 Table: Summary of necrotic cells observed after different treatment points during Triple staining of cells with Hoechst 33342 (HO), Propidium Iodide (PI) and Fluorescein diacetate (FDA). Data from this table was used to plot Fig 6B.

Treatment	% Necrotic cells at 6 hours	% Necrotic cells at 12 hours	% Necrotic cells at 24 hours	% Necrotic cells at 48 hours
**DMSO**	4.66194	5.3	4.53694	5.7
**KU 5uM**	4.66194	10.7	10.4	10.7
**KU 10uM**	5.145528	18.71	15.06854	4.3
**ETO 20uM**	5.525	9.3	9.050628	29.3
**KU5uM+ETO20uM**	8.899733	10.1	8.616239	5.1
