# Supplementary material for: Increased sensitivity of etoposide-treated breast cancer cells with an ATM inhibitor
Source: PLoS One. 2026 Jan 20;21(1):e0340472. doi: 10.1371/journal.pone.0340472 (PMC12818603; doi:10.1371/journal.pone.0340472)

Supplementary Figures

S1 Figure. Summary pie charts showing the frequency of all CBMN endpoints across treatment groups. (A) MNi per 1000 BN cells, **(B)** NBuds per 1000 BN cells, **(C)** NPBs per 1000 BN cells.

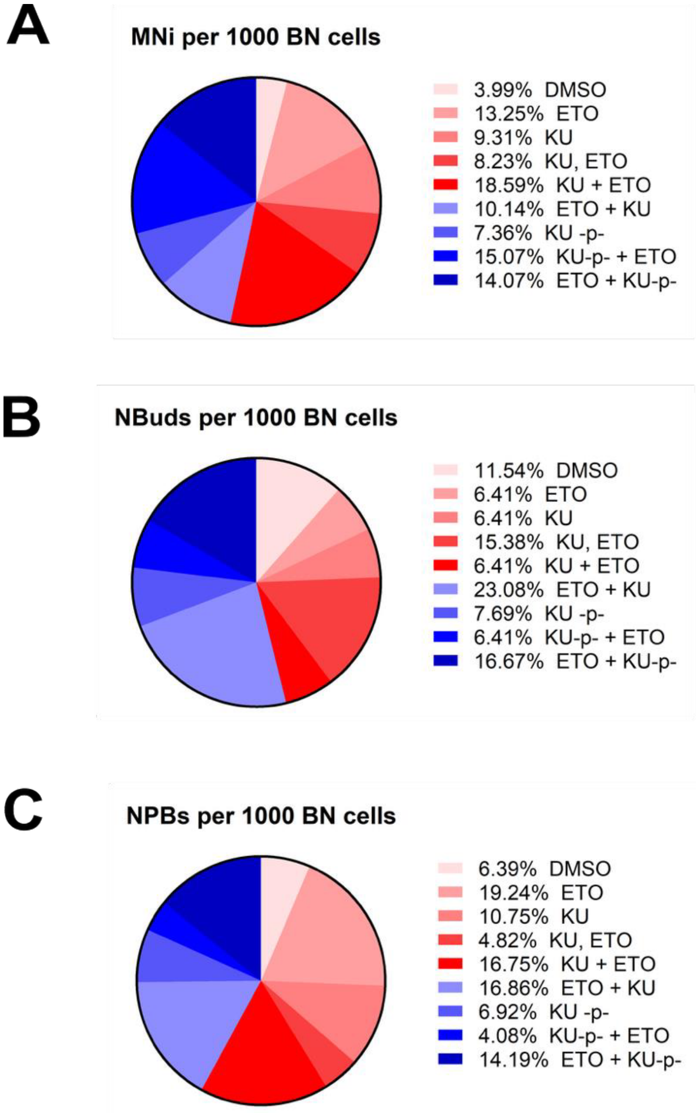

Supplement: S1 Fig — (PDF) [file pone.0340472.s005.pdf]
